# Structure and Properties of Exopolysaccharide Produced by *Gluconobacter frateurii* and Its Potential Applications

**DOI:** 10.3390/polym16071004

**Published:** 2024-04-07

**Authors:** Yingying Ning, Huiying Cao, Shouqi Zhao, Dongni Gao, Dan Zhao

**Affiliations:** 1 Engineering Research Center of Agricultural Microbiology Technology, Ministry of Education & Heilongjiang Provincial Key Laboratory of Plant Genetic Engineering and Biological Fermentation Engineering for Cold Region & Key Laboratory of Microbiology, College of Heilongjiang Province & School of Life Sciences, Heilongjiang University, Harbin 150080, China; ningyingying00@163.com (Y.N.); caohuiying1224@163.com (H.C.); zhaoshouqi1999@163.com (S.Z.); 2 Hebei Key Laboratory of Agroecological Safety, Hebei University of Environmental Engineering, Qinhuangdao 066102, China

**Keywords:** *Gluconobacter frateurii*, exopolysaccharide, purification, characterization, structure

## Abstract

An exopolysaccharide (EPS)-producing bacterium was isolated from apricot fermentation broth and identified as *Gluconobacter frateurii* HDC-08 (accession number: OK036475.1). HDC-08 EPS is a linear homopolysaccharide mainly composed of glucose linked by α-(1,6) glucoside bonds. It contains C, H, N and S elements, with a molecular weight of 4.774 × 10^6^ Da. Microscopically, it has a smooth, glossy and compact sheet structure. It is an amorphous noncrystalline substance with irregular coils. Moreover, the EPS showed surface hydrophobicity and high thermal stability with a degradation temperature of 250.76 °C. In addition, it had strong antioxidant properties against DPPH radicals, ABPS radicals, hydroxyl radicals and H_2_O_2_. The EPS exhibited high metal-chelating activity and strong emulsifying ability for soybean oil, petroleum ether and diesel oil. The milk solidification test indicated that the EPS had good potential in fermented dairy products. In general, all the results demonstrate that HDC-08 EPS has promise for commercial applications as a food additive and antioxidant.

## 1. Introduction

Microbial exopolysaccharides (EPSs) are secondary metabolites secreted by microorganisms during their growth and metabolism; they are natural polymers that can be biodegraded [1]. EPSs can be obtained by organic solvent precipitation, ultrafiltration, spray drying and freeze drying. All bacterial EPSs have been classified into two groups: homopolysaccharides (HoPSs), composed of a single type of sugar monomer, and heteropolysaccharides (HePSs), composed of two or more [2]. A large number of studies have indicated that microbial EPSs have antioxidant, antitumor, anti-inflammatory, hypoglycemic, emulsifying, flocculating and other biological activities [3]. In addition, microbial EPSs are not only natural compounds but also renewable and sustainable resources that can be applied in a very wide range. At present, a wide variety of lactic acid bacteria species have been reported to produce EPSs, such as *Lactobacillus* sp., *Weissella* sp., *Leuconostoc* sp. and *Limosilactobacillus* sp. [4,5,6]. There are also a few reports on *Enterococcus* sp., *Bacillus* sp., *Gluconobacter* sp. and *Mucor* sp. [7,8]. Therefore, the physiological metabolism of lactic acid bacteria has been studied more comprehensively [5], while there are relatively few studies on *Gluconobacter* EPSs.

*Gluconobacter* is an important genus of *Acetobacteraceae* that was first discovered by Marshall et al. [9]. Like *Acetobacter*, *Gluconobacter* is one of the core genera of *Acetobacteraneae* that was first discovered and studied. *Gluconobacter* spp. include *G. albidus*, *G. cerinus*, *G. cerevisiae*, *G. frateurii*, *G. japonicuss*, *G. kanchanaburiensis* and *G. Kondonii* [10,11]. *Gluconobacter* is widely found in the environment. Although this group is associated with spoilage in some foods, it still has great commercial value [12]. The EPSs produced by metabolism are safe and nonpathogenic to humans and animals. They are mainly used in the development of biochemical products, food additives and rapid detection and have a wide range of application prospects [13]. Due to the large differences in the structure of different *Gluconobacter* EPSs, their structural differences will inevitably lead to different biological activities. The molecular weight, monosaccharide composition and glycoside bond types of EPSs all affect the properties and biological functions of EPSs, and structural analysis and structure–activity relationship studies have become difficult topics at present [14]. However, there are few in-depth studies concentrating on *Gluconobacter* EPSs, and it is insufficient to comprehensively analyze their characteristics and application potential. Therefore, isolating and purifying more new EPS-producing *Gluconobacter*, exploring the structure and properties of EPSs and developing the functional characteristics of EPSs are the focus of current research.

In this study, an EPS produced by *G. frateurii* HDC-08 was isolated from apricot fermentation broth. The strain was identified according to morphological characteristics, 16S rRNA and sequence analysis. To gain a deeper understanding of the polysaccharide’s structure and function, HDC-08 EPS was purified and characterized, including in terms of its structure, emulsifying ability, antioxidant activity and other biochemical properties. The results of this study not only enriched the source of EPS-producing *G. frateurii* but also explored its application potential in various food industries.

## 2. Materials and Methods

### 2.1. Isolation of Bacterial Strain

Picked apricots were mashed, and 25 g of the mashed apricots was accurately weighed, mixed in 225 mL sterilized water and incubated at 30 °C for 24 h. After that, a sample solution with 10^−1^ dilution was prepared by vortex mixing. The appropriate diluent (1 mL) was poured into solid de Man, Rogosa and Sharpe medium (MRS) plates [4] and incubated in incubators (30 °C, 48 h). Single colonies with calcium-soluble rings and thick colonies were selected and then cultured and purified. The acquired single colonies were selected and cultured on MRS medium and incubated at 30 °C in a 140 r/min shake flask for 18 h. The activated strain was strewed on MRS agar medium and MRS–sucrose (MRS-S) agar medium [15] overnight culture at 30 °C. Single colonies with obvious characteristics were selected to observe colony morphology, color, size, eminence state, edge uniformity and viscosity.

### 2.2. 16S rRNA Identification of Strains

A bacterial genome extraction kit was used to extract genomic RNA. Primers 5′-TACGGYTACCTTGTTACGACTT-3′ and 5′-AGAGTTTGATCMTGGCTCAG-3′ were used for PCR amplification. The sequences were compared with existing nucleotide sequences in the GenBank database to determine their homology and submitted to the NCBI database. The nucleotide sequences of model strains with high homology of 16S rRNA nucleotide sequences were downloaded and obtained, a phylogenetic tree was constructed and the species level of strains was identified according to the homology.

### 2.3. Extraction and Purification of HDC-08 EPS

The purification of the EPS was conducted according to the method of Zhao et al. [16]. HDC-08 (1% (*v*/*v*)) was cultured in 250 mL/500 mL EPS-producing medium at 30 °C and 120 rpm for 36 h to obtain fermentation broth and then centrifuged at 8000× *g* at 4 °C for 30 min. A triploid volume of precooled 95% ethanol was added to the supernatant, which was kept overnight at 4 °C. Then, after centrifugation (8000× *g*, 30 min, 4 °C), the EPS precipitate was completely dissolved in 100 mL distilled water. An equivalent volume of 10% (*w*/*w*) trichloroacetic acid (TCA) was added to precipitate the protein, which was kept at 4 °C for 10 h. After centrifugation (8000× *g*, 45 min, 4 °C), three-fold the volume cooled with 95% ethanol was added, and the precipitate was dissolved in deionized water. This was followed by transfer to a dialysis bag (molecular weight cut-off, 14,000 Da) and dialysis at 4 °C for 2 days. After further purification by gel filtration chromatography (Sephadex G-100) and lyophilization, pure EPS (P-EPS) was obtained.

The purity of the EPS solution was measured by an ultraviolet spectrophotometer (SP-1920UV, Spectrum Instruments, Shanghai, China) in the wavelength range of 190–400 nm; nucleic acid and protein contamination were detected, thus identifying the purity of the EPS.

### 2.4. Structural Properties of HDC-08 EPS

#### 2.4.1. Determination of Molecular Mass and Elemental Composition

According to the method of Liu et al. [15], EPS (2.0 mg) was mixed with 1 mL 0.1 mol/L NaNO_3_ solution and treated with ultrasound. After centrifugation at 8000× *g* for 5 min, 10 μL of supernatant was collected, and its molecular weight was determined by gel permeation chromatography (GPC) (TDA305, Malvern, PA, USA) with a refractive index detector (RID-20A). The column temperature was 40 °C, and a TSK-Gel G4000PW_XL_ column was used. As the eluent, 0.1 mol/L NaNO_3_ solution was used at a flow rate of 0.5 mL/min. Dextran with different molecular weights was used to construct a calibration curve.

The contents of C, H, N and S in the HDC-08 EPS were determined by an elemental analyzer (Vario Micro cube, Elementar, Langenselbod, Germany). Approximately 2 mg of EPS sample was taken, wrapped, pressed and folded with tin foil. The packaged samples were put into an element analyzer under the CHNS operating mode.

#### 2.4.2. Monosaccharide Composition

Referring to the method of Dai et al. [17], HDC-08 EPS (2.0 mg) was hydrolyzed with anhydrous methanol containing 1 mol/L HCl at 80 °C for 16 h and then hydrolyzed with 2 mol/L trifluoroacetic acid (TFA) at 120 °C for 1 h. The hydrolyzed product was derived by 1-phenyl-3-methyl-5-pyrazolone (PMP). The 10 μL PMP-derived EPS sample was injected into the high-performance liquid chromatography (HPLC) system with a COSMOSIL 5C18-PAQ (4.6 ID × 250 mm) connected to a Shimadzu LC-20AT, SPD-20A UV–Vis Detector at 35 °C. The mobile phase contained 80% phosphate-buffered saline (PBS, 0.1 mol/L, pH 7.0) and 19% acetonitrile. Glucose, mannose, rhamnose, galactose and arabinose were used as the standard substances for detection at a UV absorbance of 245 nm.

#### 2.4.3. Fourier Transform Infrared Spectroscopy (FT-IR)

The FT-IR spectrum of HDC-08 EPS was recorded using a Bio-Rad IR spectrometer (Bruker, Karlsruhe, Germany). P-EPS powder was mixed with KBr at a ratio of 1:100 and then pressed into thin slices [6]. The samples were scanned in the scanning range of 4000–400 cm^−1^ with a detector resolution of 4 cm^−1^.

#### 2.4.4. Nuclear Magnetic Resonance (NMR) Spectroscopy

The ^1^H NMR, ^13^C NMR, correlation spectroscopy Y (COSY) and heteronuclear singular quantum correlation (HSQC) spectra of HDC-08 EPS were obtained by 600 MHz NMR spectroscopy (Avance III, Bruker, Karlsruhe, Germany) at 25 °C. The purified EPS (30 mg) was fully dissolved in 550 μL D_2_O and placed in NMR tubes [18]. D_2_O was used as the internal standard, and the relaxation waiting time was 1.5 s.

#### 2.4.5. Morphological Analysis

The macroscopic surface morphology of the EPS was determined by scanning electron microscopy (SEM) (S-4000, Zeiss, Jena, Germany) under a voltage of 15 kV and magnification of 400× and 1000× after the samples were adhered to conductive glue and gilded [8].

#### 2.4.6. X-ray Diffraction (XRD)

The crystalline properties of the EPS were observed by XRD (SmartLab, Tokyo, Japan). The sample powder was uniformly placed in the sample hole at a rate of 2°/min, and a scanning range of 5°–90° was used to obtain the diffraction patterns.

#### 2.4.7. Congo Red Test

The conformational structure of the EPS was detected by Congo red analysis [19]. HDC-08 EPS solution (2 mg/mL) was dissolved in an equal volume of 80 μM Congo red solution, and then different volumes of 1 mol/L NaOH solution were added for gradient dilution to achieve the final concentration (0–0.5 M). The maximum absorption wavelength (λ max) under different NaOH concentrations in the range of 400–800 nm was measured. The spatial conformation of the EPS was analyzed according to the change in λ max.

### 2.5. Water Contact Angle Analysis of HDC-08 EPS

According to Zhao et al.’s method [20], HDC-08 was inoculated in MRS and MRS-S medium containing 5% sucrose for 48 h, centrifuged at 4000× *g* for 10 min, and air-dried at room temperature. The data were detected by an OCA20 contact angle analyzer (LAUDA Scientific, Lauda-Königshofen, Germany).

### 2.6. Antioxidant Activity Tests of HDC-08 EPS

#### 2.6.1. 1,1-Diphenyl-2-picrylhydrazyl (DPPH) Radical Scavenging Assay

Based on Pei et al.’s method [21], a 2 mL (concentration range 0–5 mg/mL) EPS sample was combined with 2 mL 0.2 mmol/L DPPH–ethanol solution. After the reaction proceeded in the dark for 30 min, the absorbance at *OD*_517nm_ of the mixed solution was determined with ascorbic acid (Vc) as a positive control.

#### 2.6.2. 2,2′-Azino-bis-(3-ethylbenzthiazoline-6-sulfonate) (ABTS) Radical Scavenging Assay

According to the method of Jiang et al. [22], HDC-08 EPS (concentration range 0–5 mg/mL, 2 mL) was combined with 4 mL of 7 mmol/L ABTS working solution, and the reaction was carried out at room temperature for 6 min. The absorbance value at *OD*_734nm_ was determined.

#### 2.6.3. Hydroxyl Radical Scavenging Assay

As reported by Tang et al. [23], HDC-08 EPS (concentration range 0–5 mg/mL, 2 mL) was mixed with 1 mL of FeSO_4_ solution (9 mmol/L) and 1 mL of salicylic acid–ethanol solution (9 mmol/L). H_2_O_2_ solution (1 mL 9 mmol/L) was added and reacted at 37 °C for 40 min. The absorbance value at *OD*_734nm_ was determined.

#### 2.6.4. H_2_O_2_ Scavenging Assay

H_2_O_2_ scavenging activity was determined according to the modified method of Bukola et al. [24] with some adjustments. A total of 0.6 mL of 40 mmol/L H_2_O_2_ solution was mixed with 2.4 mL of 0.1 mol/L pH 7.4 phosphate buffer, and 2 mL (concentration range 0–5 mg/mL) of HDC-08 EPS was added to it. The reaction system was mixed and reacted for 10 min. The mixed solution was measured at *OD*_230nm_.

### 2.7. Milk Solidification Test

The strain HDC-08 was inoculated into 10% (*w*/*v*) sterile skim milk with the inoculum of 1% (*v*/*v*), with the addition of 0, 3, 6, 9 or 12% (*w*/*v*) sucrose. This was followed by stationary culture for 24, 36 and 48 h at 30 °C. Skim milk with 0% sucrose was used as a negative control.

### 2.8. Metal-Chelating Activity of HDC-08 EPS

Two milliliters of 10 mg/mL HDC-08 EPS solution was added to 50 mL of 10 mg/L different metal solutions (Cu^2+^, Zn^2+^ and Fe^2+^) [21]. The mixed solution was then incubated at 26 °C for 2 h and separated by centrifugation at 10,000× *g* for 10 min. The residual metal concentrations were measured by an atomic absorption spectrometer (iCE 3500 Thermo Scientific, Waltham, MA, USA).

### 2.9. Thermodynamic Properties of HDC-08 EPS

A thermal analysis instrument (STA449f3, Netzsch, Stuttgart, Germany) was used for differential scanning calorimetry (DSC), thermogravimetric analysis (TGA), and differential thermogravimetry (DTG) evaluation of HDC-08 EPS. HDC-08 EPS powder (4 mg) was added into an Al_2_O_3_ crucible with an Ar gas flow rate of 50 mL/min, and the EPS sample was heated from 25 °C to 800 °C at a heating rate of 10 °C/min [25]. The changes in the properties of the EPS with increasing temperature were recorded.

### 2.10. Viscosity of HDC-08 EPS

The intrinsic viscosity [*η*] (1/[*c*], mL/g) of HDC-08 EPS was determined by a Uttner viscometer (1834, Mitong, Shanghai, China) [22]. At 25 °C and 35 °C, the intrinsic viscosity of the EPS solution [*η*] was calculated according to Formulas (1)–(4). Each experiment was repeated three times; the constants k′ and k″ are related to the EPS and solvent.

(1) $$\eta_{r}=\frac{t}{t_{0}}$$

(2) $$\eta_{sp}=\eta_{r}-1$$

Huggins formula:

(3) $$\frac{\eta_{sp}}{c}=\left[\eta \right]+k^{\prime }{\left[\eta \right]}^{2}c$$

Kraemer formula:

(4) $$ln\frac{\eta_{r}}{c}=\left[\eta \right]+k^{\prime \prime }{\left[\eta \right]}^{2}c$$

where t is the outflow time of the EPS solution, t_0_ is the outflow time of the solvent, *η_r_* is the relative viscosity of the EPS solution, and *η_sp_* is the increased viscosity of the EPS solution.

### 2.11. Emulsifying Capacity of HDC-08 EPS

The purified HDC-08 EPS solution (2.5 mL, 1.0 mg/mL) was added to 2.5 mL of gasoline, diesel oil, soybean oil, hexane, benzene, xylene, petroleum ether and ether, followed by vortexing vigorously to homogeneity for 2 min. The height of the emulsion layer was measured by the emulsion with Vernier calipers at 0, 24, 48 and 72 h. The calculation formula is as follows:

EA (%) = (H_1_/H_2_) × 100 (5)

where H_1_ is the emulsion height (mm) and H_2_ is the mixture height (mm).

### 2.12. Statistical Analysis

All results are repetitions of at least three measurements, and the data are presented as the mean ± standard deviation (SD). JMP software (version 9.0.2, NC, SAS Institute Inc., Cary, NC, USA) was used for the statistical analysis of the data. Statistical analysis of variance (ANOVA) and Tukey’s test were considered to detect statistical significance (*p* < 0.05).

## 3. Results

### 3.1. Strain Isolation and Identification

**Figure 1:**
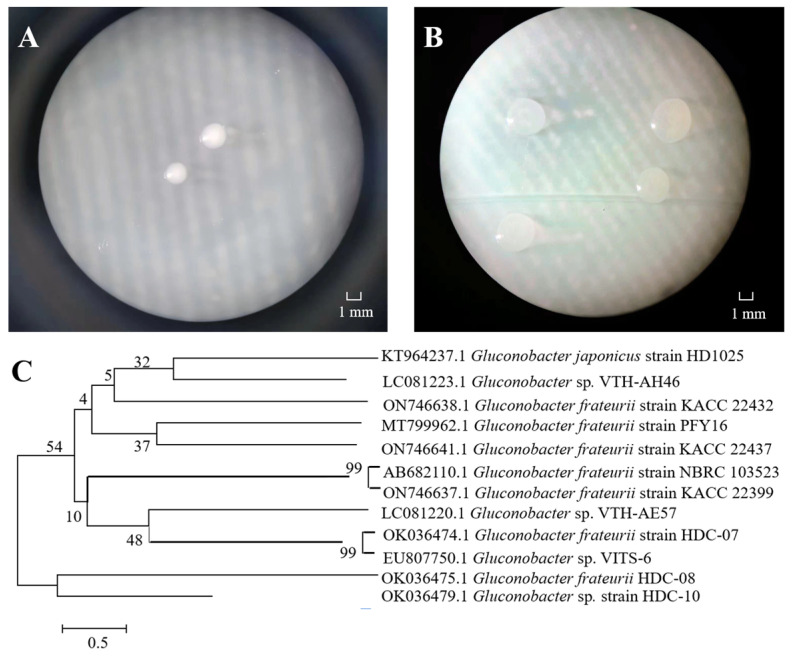
The colony morphology of strain HDC-08 on MRS (**A**) and MRS-S (**B**) agar plate at 10×; phylogenetic tree of strain HDC-08 by Neighbor-Joining method (**C**).

One EPS-producing bacterium strain was isolated from an apricot fermentation broth, forming white, round, opaque and smooth colonies on MRS agar (Figure 1A). The colony was larger on MRS-S, and a sticky liquid was attached to the surface (Figure 1B). Yukphan et al. [26] isolated *G. aidae* from tropical fruits in Thailand and observed similar colony characteristics. The 16S rRNA sequence revealed that the length of the strain was 1376 bp, and NCBI database comparison verified that the strain was closely related to several *Gluconobacter frateurii* gene sequences. There was 99% homology with *G. frateurii* HDC-07 (Figure 1C). Therefore, HDC-08 was identified as *G. frateurii* and named *G. frateurii* HDC-08. The sequence was submitted to the NCBI database, and the accession number is OK036475.1.

### 3.2. Purification and Elemental Analysis of HDC-08 EPS

**Figure 2:**
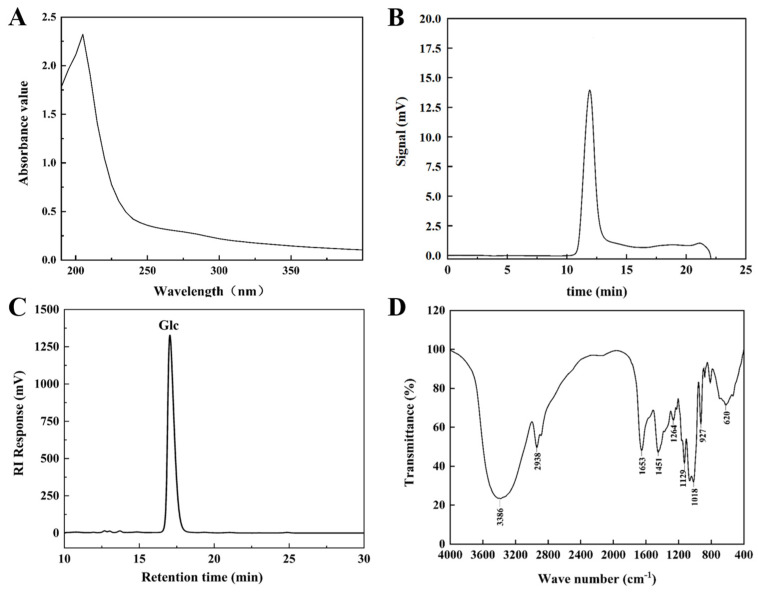
Ultraviolet spectrum (**A**), GPC (**B**), HPLC (**C**), FT-IR spectrum (**D**) of *G. frateurii* HDC-08 EPS.

After full wavelength scanning of the EPS solution, no absorption peak appeared at 260 nm and 280 nm (Figure 2A), suggesting the absence of nucleic acid and protein contamination in the EPS solution. The elemental composition analysis demonstrated that HDC-08 EPS contained 39.35% C, 6.26% H, 0.081% S and 0.21% N. C reached the highest level, followed by H, indicating that it was mainly composed of sugars. The C and H content of the EPS was highly in agreement with the EPS produced by *Leuconostoc citreum* B-2 (39.05% and 7.19%) [27]. Compared with *Saccharomyces cerevisiae* Y3 EPS (2.62%) [15], the lower content of N element in HDC-08 EPS was due to a low amount of protein modifications.

### 3.3. Structural Properties of HDC-08 EPS

#### 3.3.1. Molecular Weight Determination for HDC-08 EPS

The GPC spectrum of HDC-08 EPS is exhibited in Figure 2B. The eluting peak showed a single symmetric peak, which once again verified that the EPS was highly homogeneous. The molecular weight of the EPS was calculated as 4.774 × 10^6^ Da based on the standard curve (log Mw = −0.222x + 9.321, R^2^ = 0.9994). In general, the molecular weight of microbial EPS is mostly between 4.0 × 10^4^ Da and 6.0 × 10^6^ Da [28]. The molecular weight of HDC-08 EPS was higher than that of *Weissella cibaria* SJ14 EPS (7.12 × 10^4^ Da) [29] but was lower than that of *Leu. pseudomesenteroides* DRP-5 EPS (6.23 × 10^6^ Da) [30]. In comparison to low-molecular-weight EPSs, high-molecular-weight EPSs exhibit better solubility and stability, allowing them to function more effectively within organisms. The molecular weight of EPSs varied among different strains, potentially influenced by fermentation time and the composition of the medium.

#### 3.3.2. GPC Analysis for HDC-08 EPS

The monosaccharide composition of HDC-08 EPS was measured by HPLC (Figure 2C), revealing it was a homopolysaccharide composed of glucose units. This result was in agreement with *W. cibaria* YB-1 dextran [31]. However, other reports have shown that EPSs contain more than only glucose. For example, Salomon [32] discovered that *Cryptococcus flavescens* EPS was composed of mannose (78.32%), galactose (6.24%) and glucose (11.81%). The EPS showed exceptional viscosity and strong cholesterol-lowering abilities. In general, EPSs can be composed of glucose, galactose, mannose, rhamnose, arabinose and other monosaccharides [33]. Different types of microorganisms may have different monosaccharide compositions, which also determine the characteristics and applications of EPSs. It has been reported that mannose had good antitumor properties and could enhance the phagocytosis ability of macrophages [2].

#### 3.3.3. FT-IR Analysis for HDC-08 EPS

The FT-IR spectrum of HDC-08 EPS in the spectral range 4000–400 cm^−1^ is displayed in Figure 2D. At 3386 cm^−1^, there was a wide and strong absorption peak generated by O-H bond stretching vibrations [31]. This region was a specific signal of polysaccharides. A weak band at 2938 cm^−1^ was associated with the stretching vibration of the C-H bond [34]. In addition, the peaks at 1653 cm^−1^ and 1451 cm^−1^ indicated C=O and C-O bond stretching vibrations, respectively [35]. The spectral region of 950–1200 cm^−1^ was the EPS fingerprint, which can reflect the specificity of different carbohydrate structures and judge a specific polysaccharide by the intensity and position of specific spectral peaks [21]. Moreover, the peak at 1018 cm^−1^ verified the presence of *α*-(1,6) glucoside linkages in HDC-08 EPS, and the bending vibration at 927 cm^−1^ was associated with the pyranose configuration [4].

#### 3.3.4. NMR Spectroscopy Analysis

**Figure 3:**
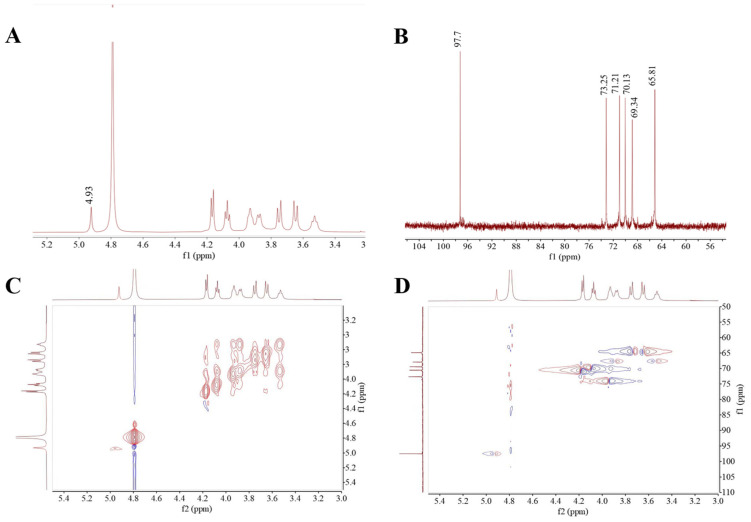
^1^H (**A**),^13^C (**B**), COSY (**C**), HSQC, (**D**) NMR spectra of *G. frateurii* HDC-08 EPS.

NMR is one of the tools for the analysis of glycosidic linkage and substituent position in EPS. The ^1^H NMR spectrum featured the anomeric proton regions (δ 4.5–5.5 ppm), the ring proton regions of C2–C6 (δ 3.1–4.5 ppm) and the alkyl signal region (δ 1.2–2.3 ppm) [36]. As exhibited in Figure 3A, the HDC-08 EPS sample had a distinct signal peak at δ 4.93 ppm, corresponding to *α*-(1,6)-linked glucan with the D-pyranose residue, which was consistent with the FT-IR results. The ^13^C NMR spectrum (Figure 3B) could be used to identify the anomeric carbons (resonate between 95 and 110 ppm) and the nonanomeric carbons (resonate between 50 and 85 ppm) [37]. The absence of a peak at δ101–105 ppm indicated that the glycosidic bond in HDC-08 EPS had an *α*-configuration. Moreover, there was no peak at δ 90 ppm, which was the signal of the EPS that had only a pyranose configuration. The signal peaks at δ 73.25, 71.21, 70.13, 69.34 and 65.81 ppm in the ^13^C NMR spectrum correspond to the signal peaks of C3, C2, C5, C4 and C6 in other unconnected pyranose rings, respectively.

Two-dimensional NMR approaches, such as COSY and HSQC, can be used to determine the correlations between different carbon and hydrogen atoms in EPS, thus further determining the structure of the EPS. The COSY spectral diagram of HDC-08 EPS is shown in Figure 3C, and the signal peak at δ 4.93 ppm was the characteristic signal peak of H-1. The HSQC atlas can be used to analyze the correlation between each 1H and its directly connected ^13^C (Figure 3D). The main chain of HDC-08 EPS is *α*-(1,6) glycosidic bonds (4.93/97.7 (H1/C1), 4.16/71.21 (H2/C2), 3.92/73.25 (H3/C3), 3.54/69.34 (H4/C4), 4.07/70.13 (H5/C5) and 3.76, 3.64/65.81 (H6, H6′/C6)). In general, the NMR data showed that the structure of HDC-08 EPS was a linear *α*-(1,6)-linked dextran, which was similar to the EPS from *W. confusa* XG-3 [20].

#### 3.3.5. Microstructure of HDC-08 EPS

**Figure 4:**
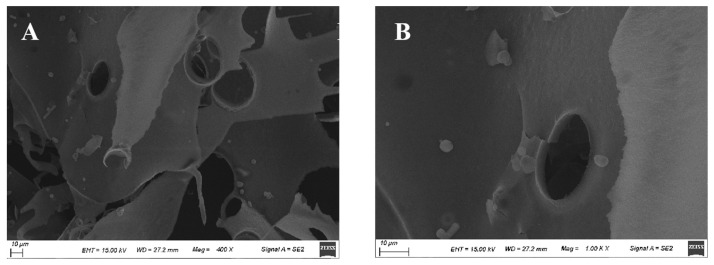
Surface morphology of *G. frateurii* HDC-08 EPS at various magnifications: 400× (**A**), 1000× (**B**).

Based on SEM observations of the surface morphology, the physical properties of polysaccharides can be inferred, providing a theoretical basis for a deeper understanding of their biological activity. As shown in Figure 4, HDC-08 EPS exhibited a smooth, glossy and compact sheet structure. The compact structure of the sheet gave the EPS good mechanical stability. Smooth and glossy properties enable EPSs to be prepared into plastic film materials, which can be used in food and cosmetic fields [38]. The SEM results of *Paenibacillus polymyxa* 92 EPS showed that the EPS was smooth and presented a thin plate shape [39], which was in agreement with the results of this study. The microscopic results of the EPS were closely related to the source of the strain and the culture conditions of EPS production.

#### 3.3.6. X-ray Diffraction Analysis

**Figure 5:**
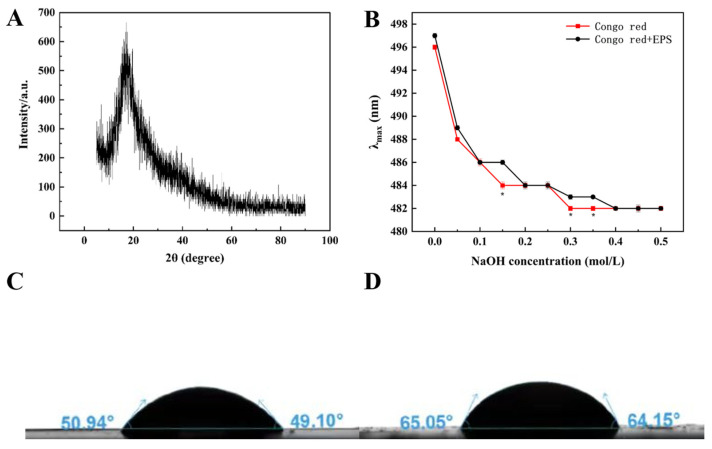
The XRD spectra of *G. frateurii* HDC-08 EPS (**A**); plots of the λmax of Congo red and Congo red + *G. frateurii* HDC-08 EPS in solutions with various concentrations of NaOH (**B**); water contact angle analysis of *G. frateurii* HDC-08 in MRS (**C**) and MRS-S (**D**). *p* < 0.05 (*) indicates significant differences.

The crystal structure of an EPS is closely related to solubility, expansibility and some physical properties. XRD spectra can be used to effectively analyze the amorphous or crystalline characteristics of EPSs. HDC-08 EPS displayed an amorphous noncrystalline substance, with strong diffraction peaks occurring only when 2θ was 20° (Figure 5A). This was because the EPS contained many amorphous regions, and a few crystal regions were hidden in the amorphous regions, presenting a relatively amorphous state as a whole. Similar studies also indicated that *Leu. lactis* L2 EPS [16] and *W. confusa* C19 EPS [40] had an amorphous noncrystalline nature.

#### 3.3.7. Triple-Helical Structure Analysis

Congo red is a commonly used method to detect whether an EPS contains a triple-helical structure. Congo red can form a complex with triple-helix EPS, and the complex will undergo a bathochromic shift in a weakly alkaline solution. As shown in Figure 5B, HDC-08 EPS and the Congo red complex had no notable bathochromic shift, suggesting that the form of HDC-08 EPS was an irregular coil without a triple-helical structure. *W. confusa* XG-3 EPS [20] and *Lactobacillus sakei* L3 EPS [41] also exhibited irregular coil conformations. Studies have indicated that low-molecular-weight EPSs cannot form a triple-helical structure, while high-molecular-weight EPSs (>9 × 10^6^ Da) can form a triple-helical structure.

### 3.4. Biotechnological Applications

#### 3.4.1. Hydrophobicity Analysis of HDC-08 EPS

The water contact angle is an important index related to the hydrophobicity of EPSs. A smaller contact angle indicates excellent hydrophilicity. The result of HDC-08 in the MRS medium (Figure 5C) was 50.94°, while that in the MRS-S medium (Figure 5D) containing 5% sucrose was 65.05°, suggesting that sucrose significantly induced the production of EPS by HDC-08. This result was similar to the contact angle of *Lac. sakei* L3 [42] but was different from that of *W. cibaria* 27 [43].

#### 3.4.2. Antioxidant Activity Analysis of HDC-08 EPS

**Figure 6:**
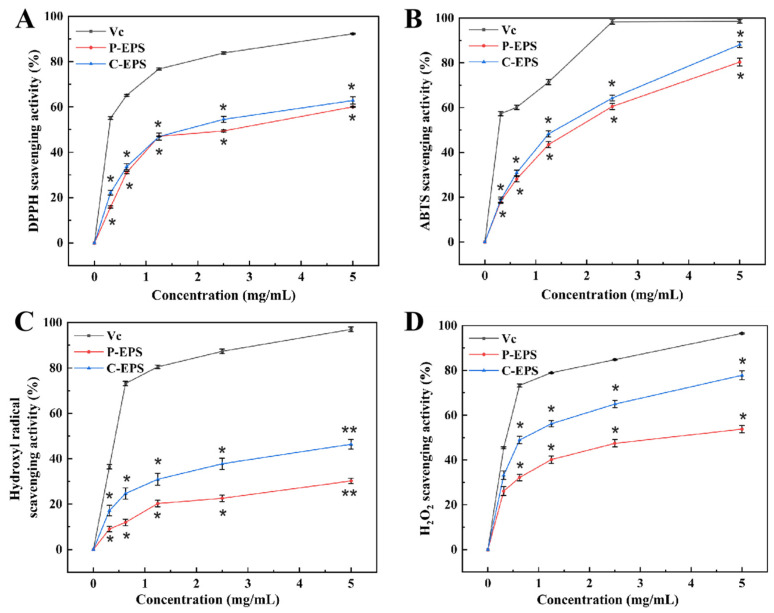
The DPPH (**A**), ABTS (**B**), hydroxyl radical (**C**) and H_2_O_2_ (**D**) scavenging activity of *G. frateurii* HDC-08 EPS. *p* < 0.05 (*) and *p* < 0.01 (**) indicate significant differences.

DPPH is a significant reagent used to measure antioxidant properties; it can transfer electrons to radicals and neutralize the properties of radicals, resulting in the yellow color of a DPPH solution [44]. As shown in Figure 6A, the tested samples all exhibited a certain enhancement effect with increasing concentration. The highest scavenging rates of HDC-08 P-EPS and C-EPS were 60.05 ± 2.44% and 62.86 ± 1.25% at 5.0 mg/mL, respectively. These results were higher than the levels of C-EPS (45.26 ± 3.5%) and P-EPS (42.76 ± 2.0%) produced from *W. confusa* XG-3 [20] at the same concentration. The existence of functional groups such as -O-, C=O and -OH could supply electrons or hydrogen to DPPH radicals, significantly reducing free radicals.

As displayed in Figure 6B, the ABTS radical scavenging ability of C-EPS was always higher than that of P-EPS and increased with the EPS concentration. The scavenging abilities of P-EPS and C-EPS were 80.32 ± 1.67% and 88.12 ± 0.82% when the concentration reached 5.0 mg/mL. The study of Elmansy et al. [45] showed that *Lac. plantarum* RO30 EPS had antioxidant activity when the EPS concentration reached 5 mg/mL; the ABTS radical clearance rate was 49.1% ± 1.32%. *Pediococcus pentosaceus* M41 EPS [46] also exhibited a good ABTS radical scavenging rate (48.97 ± 0.89%). However, the scavenging activity of both was lower than that of HDC-08 EPS. Different strains of EPSs showed different scavenging abilities for ABTS radicals, which may be associated with the different compositions and functional groups of EPSs.

Hydroxyl radicals are thought to have strong oxidizability and could harm biological tissue, inducing severe damage [47]. At 5.0 mg/mL, the hydroxyl radical scavenging ability of P-EPS and C-EPS reached 30.22 ± 0.86% and 46.39 ± 0.72%, respectively (Figure 6C). Compared with *Leu. citreum* B-2 EPS (12.9 ± 0.14%) [48], the results confirmed that HDC-08 EPS showed a stronger ability to scavenge.

H_2_O_2_ can indirectly produce hydroxyl radicals in cells, inducing severe oxidative damage. As displayed in Figure 6D, the H_2_O_2_ scavenging ability was gradually enhanced with increasing HDC-08 EPS and reached the highest value (C-EPS (77.8 ± 2.30%)) at a concentration of 5 mg/mL. HDC-08 EPS presented good H_2_O_2_ scavenging ability, but it was slightly weaker than that of the EPS from *W. confusa* (81.0 ± 1.09%) [24]. Many studies have reported that the antioxidant activity of EPSs in vitro was associated with the monosaccharide composition [39]. Additionally, when an EPS has substituents such as sulfate, acetate and phosphate groups, it can further expose more hemiacetal hydroxyl groups, enhancing the antioxidant activity of polysaccharides.

#### 3.4.3. Coagulation Effect of HDC-08 EPS on Skim Milk

**Figure 7:**
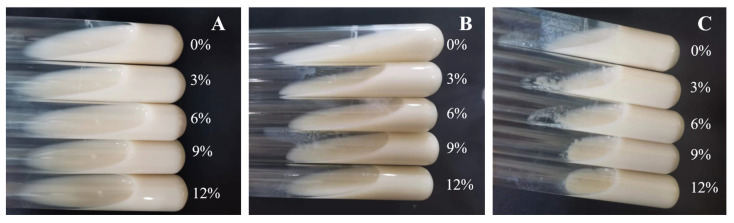
Coagulation effect of *G. frateurii* HDC-08 on 10% skim milk at 24 h (**A**), 36 h (**B**) and 48 h (**C**).

As shown in Figure 7, the presence of sucrose demonstrated a certain degree of coagulation, and the coagulation effect became stronger with the increase in sucrose concentration and culture time. When cultured for 48 h in the presence of 12% sucrose, the skim milk achieved a complete curdling effect. The results indicated that sucrose could significantly promote the production of EPSs. Wang et al. [42]. and Ayyash et al. [49] also obtained the same result. In short, HDC-08 EPS had good application potential in fermented dairy products.

#### 3.4.4. Metal-Chelating Activity of HDC-08 EPS

**Figure 8:**
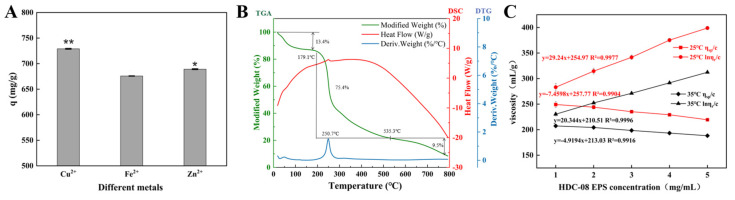
Metal adsorption activity (**A**), thermogravimetry (**B**) and viscosity characteristic (**C**) of *G. frateurii* HDC-08 EPS. *p* < 0.05 (*) and *p* < 0.01 (**) indicate significant differences.

Metal-resistant bacteria and their EPSs are powerful in removing metals from wastewater and the environment [50]. The HDC-08 EPS had a comparatively high affinity towards Cu^2+^ and Zn^2+^, with q values of 728.99 ± 0.75 mg/g and 689.15 ± 0.78 mg/g, respectively (Figure 8A). In contrast, the adsorption efficiency of the EPS on Fe^2+^ was lower, and the q value was 675.77 ± 0.35 mg/g. The adsorption capacity of HDC-08 for Cu^2+^ and Zn^2+^ was significantly higher than that of *Athelia rolfsii* [51], and its q values were 480.9 ± 57.81 mg/g and 159.6 ± 21.06 mg/g, respectively. The existence of functional groups such as -O- and C=O can effectively bind metal ions [21].

#### 3.4.5. Characterization of Thermal Properties

The thermodynamic analysis of HDC-08 EPS is displayed in Figure 8B. The TGA curve indicated that the EPS dehydrated and cracked with increasing temperature. There were three main stages to this process. In the first stage (18.6 °C–173.2 °C), the sample mass loss was 13.4% due to the loss of free water, suggesting that a large number of carboxyl groups in HDC-08 EPS combined with water molecules. In the second stage (179.1 °C to 535.3 °C), heat loss was significant (reduced by approximately 75.4%), which was attributed to the degradation of the EPS. After this, the weight of the polysaccharide sample gradually decreased until it reached a constant value (with a mass loss of 9.5%).

The DTG curve presented a sharp peak, suggesting that the weight loss rate reached a maximum at 250.7 °C (Figure 8B). As a result, the degradation temperature of HDC-08 EPS was 250.7 °C. This result was slightly lower than that of *W. confusa* MD1 EPS (267.7 °C) [52] and *W. confusa* KR780676 EPS (287.5 °C) [53]. As shown in Figure 8B, there was an endothermic peak at 251.2 °C, which can be associated with the melting of crystals formed by long fatty side chains during the depolymerization of the principal component of EPS samples. This result was in line with the TGA and DTG curves. In conclusion, HDC-08 EPS had good thermal stability and could be safely used in food applications.

#### 3.4.6. Viscosity Analysis of HDC-08 EPS

The intrinsic viscosity of EPSs mainly depends on their molecular weight and structure. In general, an EPS with a higher molecular weight can more effectively form a denser network structure with the solvent. The viscosity of HDC-08 EPS at 25 °C and 35 °C is depicted in Figure 8C. According to the curve equation, the viscosity of EPSs at the intersection of the Y-axis was 256.95 mL/g and 217.54 mL/g, respectively. The results verify that the viscosity is negatively correlated with temperature. The viscosity of HDC-08 EPS was lower than that of bacterial EPSs, such as *W. confusa* XG-3 EPS (409.70 mL/g) [54], and lower than that of plant polysaccharides, such as galactomannan from *Astragalus gombo* (860.68 mL/g) [55]. Due to its low viscosity, HDC-08 EPS can serve as a food additive for increasing the viscosity of dairy products such as milk, yogurt and cheese, improving their texture.

#### 3.4.7. Emulsifying Properties of HDC-08 EPS

The emulsifying capacity of HDC-08 EPS for different oils (gasoline, diesel oil, soybean oil) and hydrocarbons (hexane, benzene, xylene, petroleum ether, ether) is shown in Table 1. With increasing time, the emulsifying ability of the EPS for oils and hydrocarbons was also enhanced. The emulsification value was the highest at 72 h, and the EA values for petroleum ether and soybean oil were higher than others, being 21.11 ± 0.16% and 34.80 ± 0.18%, respectively. Among the tested oils and hydrocarbons, the overall emulsification trend is soybean oil > petroleum ether > diesel oil > benzene > gasoline > xylene > ether > hexane. The results were similar to those for *W. confusa* H2 EPS [56], showing good emulsifying properties. Therefore, HDC-08 EPS can be used as an emulsifier to help stabilize oil–water mixtures, improving the texture and mouthfeel of food.

## 4. Conclusions

In this study, the EPS of *G. frateurii* HDC-08 was isolated, purified and characterized. HDC-08 EPS is a dextran composed of glucose and connected by *α*-(1,6) glucoside bonds with a molecular weight of 4.774 × 10^6^ Da. The EPS reflects a smooth, glossy and compact sheet structure, which has the potential to be used as a stabilizer and plastic film material. It showed an irregular coil structure and amorphous noncrystalline substance. In addition, EPS exhibited surface hydrophobicity, good thermal stability and antioxidant ability against DPPH radicals, ABPS radicals, hydroxyl radicals and H_2_O_2_. Good metal-chelating activity and strong emulsifying ability were observed for soybean oil, petroleum ether and diesel oil. The milk coagulation ability of HDC-08 EPS gives it good application potential in fermented dairy products. All these results imply that HDC-08 EPS has prospects for application in the fields of food.

## Figures and Tables

**Table 1:** EA% of *G. frateurii* HDC-08 EPS with organic agents.

Organic Agents	EA%			
	1 h	24 h	48 h	72 h
Hexane	2.22 ± 0.08 ^F,d^	2.72 ± 0.11 ^G,c^	3.99 ± 0.13 ^H,b^	5.99 ± 0.28 ^H,a^
Benzene	6.32 ± 0.11 ^C,d^	10.84 ± 0.10 ^C,c^	14.47 ± 0.15 ^D,b^	16.70 ± 0.13 ^D,a^
Xylene	3.93 ± 0.18 ^E,d^	8.20 ± 0.20 ^E,c^	10.32 ± 0.15 ^F,b^	12.69 ± 0.21 ^F,a^
Petroleum ether	8.47 ± 0.13 ^B,d^	13.65 ± 0.11 ^B,c^	18.74 ± 0.13 ^B,b^	21.11 ± 0.16 ^B,a^
Ether	1.96 ± 0.08 ^F,d^	5.93 ± 0.18 ^F,c^	6.81 ± 0.11 ^G,b^	8.80 ± 0.10 ^G,a^
Gasoline	4.99 ± 0.20 ^D,d^	9.54 ± 0.15 ^D,c^	12.33 ± 0.15 ^E,b^	16.18 ± 0.23 ^E,a^
Diesel oil	8.73 ± 0.10 ^B,d^	10.77 ± 0.15 ^C,c^	15.41 ± 0.20 ^C,b^	19.00 ± 0.18 ^C,a^
Soybean oil	18.48 ± 0.18 ^A,c^	27.05 ± 0.08 ^A,c^	31.21 ± 0.28 ^A,b^	34.80 ± 0.18 ^A,a^

Capital letters show significant differences in data in one column. Lowercase letters show significant differences in data in one row. *p* < 0.05 indicates significant differences.

## Data Availability

Data are contained within the article.
